# Nested synchrony—a novel cross-scale interaction among neuronal oscillations

**DOI:** 10.3389/fphys.2012.00384

**Published:** 2012-09-26

**Authors:** Simo Monto

**Affiliations:** Department of Biomedical Engineering and Computational Science, School of Science, Aalto UniversityEspoo, Finland

**Keywords:** neuronal oscillations, magnetoencephalography, nested oscillations, oscillation synchrony

## Abstract

Neuronal interactions form the basis for our brain function, and oscillations and synchrony are the principal candidates for mediating them in the cortical networks. Phase synchrony, where oscillatory neuronal ensembles directly synchronize their phases, enables precise integration between separated brain regions. However, it is unclear how neuronal interactions are dynamically coordinated in space and over time. Cross-scale effects have been proposed to be responsible for linking levels of processing hierarchy and to regulate neuronal dynamics. Most notably, nested oscillations, where the phase of a neuronal oscillation modulates the amplitude of a faster one, may locally integrate neuronal activities in distinct frequency bands. Yet, hierarchical control of inter-areal synchrony could provide a more comprehensive view to the dynamical structure of oscillatory interdependencies in the human brain. In this study, the notion of nested oscillations is extended to a cross-frequency and inter-areal model of oscillatory interactions. In this model, the phase of a slower oscillation modulates inter-areal synchrony in a higher frequency band. This would allow cross-scale integration of global interactions and, thus, offers a mechanism for binding distributed neuronal activities. We show that inter-areal phase synchrony can be modulated by the phase of a slower neuronal oscillation using magnetoencephalography (MEG). This effect is the most pronounced at frequencies below 35 Hz. Importantly, changes in oscillation amplitudes did not explain the findings. We expect that the novel cross-frequency interaction could offer new ways to understand the flexible but accurate dynamic organization of ongoing neuronal oscillations and synchrony.

## Introduction

Neurons are capable of synchronizing their activity to a collective rhythm. These neuronal oscillations vary in frequency, amplitude, and source topography (Buzsaki and Draguhn, 2004). Theoretical (Singer and Gray, 1995) and experimental (Womelsdorf et al., 2006) work converge on the idea that synchronous neuronal assemblies are central for neuronal communication. Distinct oscillatory assemblies are able to synchronize their activities, and it has been proposed that such coherent oscillations provide temporal windows for efficient communication between distinct brain regions (Fries, 2005). Indeed, cortical oscillations and synchrony have been found to regulate stimulus processing in the neuronal (Cardin et al., 2009) and behavioral (Hamidi et al., 2009) level. Furthermore, it has been shown that such oscillation synchrony is related to neuronal spiking activity (Canolty et al., 2010). Thus, oscillatory neuronal populations and their synchronization play a key role in integrating activities in single cells and in the system level.

In monkey recordings, neuronal rhythms have been shown to provide windows of increased excitability that enhance processing of rhythmic stimuli (Schroeder and Lakatos, 2009). Interestingly, several experiments have found that these oscillations are organized so that the amplitude of a higher frequency oscillation correlates with the phase of a slower rhythm (Lakatos et al., 2005). This cross-frequency model of an oscillatory interaction, phase-modulated amplitude, is called a nested oscillation. Such hierarchical organization of nested rhythmic activities has been observed in a wide frequency range in human intracranial recordings as well, and these data support the functional significance of nested oscillations by showing that experimental conditions modulate the nested relationships (Canolty et al., 2006; He et al., 2010). Also extracranially recorded magnetoencephalography (MEG) data from resting humans has previously revealed a nested interaction between alpha and gamma frequency bands (Osipova et al., 2008).

**Figure 1:**
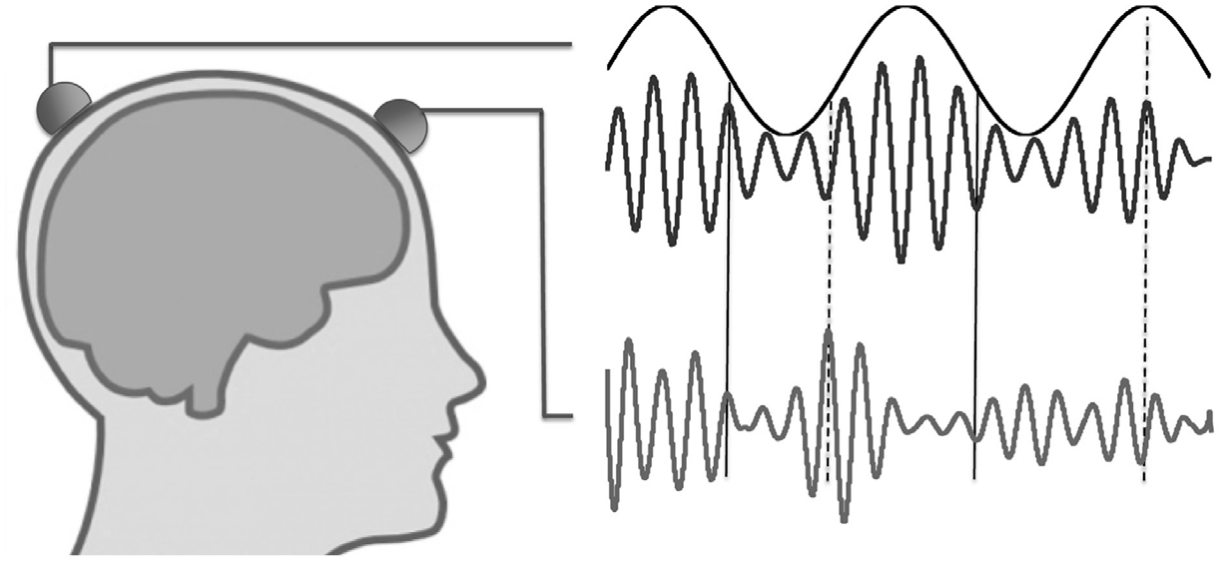
**Illustration of nested oscillations and nested synchrony.** In nested oscillations, the phase of the slower oscillation modulates the higher-frequency amplitude measured at the same scalp location (two upper traces), but not that measured at a different location (lower trace). In contrast, nested synchrony means that the two faster oscillations become more tightly coupled in certain phases of the slow oscillation (around solid vertical lines) than in other phases (around dashed vertical lines).

In theoretical accounts of nested oscillations, the low-frequency oscillation has often been associated with periodic excitability changes, which then affects the amplitude of oscillations in higher frequency bands (Jensen and Colgin, 2007; Lakatos et al., 2008). The increased oscillation amplitude, which is observed in the higher frequency band, is considered to reflect not only increased levels of synaptic or spiking activity, but also enhanced neuronal synchronization. Based on this interpretation, we suggest a novel model of a non-local cross-frequency interaction, where the phase of the slower oscillation regulates inter-areal synchrony in the higher frequency band (Figure 1). In the above context this is analogous to the model of nested oscillations, as they both are then related to phase-modulation of neuronal synchrony, albeit in different spatial scales. Our model, which we term nested synchrony, includes both a cross-frequency interaction and an inter-areal interaction. Thus, it could be one candidate for mediating the complex dynamic relationships of neuronal oscillations across time scales and brain regions. Regulation of synchrony dynamics could be achieved through coordinating inter-areal synchrony in a higher frequency band by possibly meta-stable and scale-free dynamics provided by the lower frequencies. The aim of this study is to demonstrate the presence of nested synchrony in the human brain using MEG data recorded at rest.

## Methods

### Experiment

We recorded MEG (Elekta Neuromag Oy, Finland) data from normal, consent subjects (*N* = 4; age 28–35 years, 1 female) in a silent, magnetically shielded room. The experiment was approved by the Ethical Committee of Hospital District of Helsinki and Uusimaa. The experiment consisted of one session of 20 min, during which the subjects were at rest, eyes closed. The sampling rate was 600 Hz, and the high-pass and low-pass acquisition filters were at 0.03 and 172 Hz. Data was recorded with 306 channels, of which the 204 planar gradiometer channels were used for this study.

### Pre-processing

The data was first subjected to noise reduction by spatial Signal Space Separation (SSS) filtering and temporal projection of noise components by the temporal extension of SSS (Taulu and Simola, 2006) using the MaxFilter software (Elekta Neuromag Oy, Finland). Subsequent analyses were performed with custom-made software running in Matlab (MathWorks Inc, Natick, MA, U.S.A.).

Part of the cardiac artifact was not removed by SSS, and was therefore treated by applying ICA to the data. The component(s) corresponding to heart-related activity were recognized by hand based on their temporal dynamics, and projection in temporal domain was then applied to project them away. The data was then windowed to 4 s epochs, and bad epochs were discarded if peak-to-peak amplitude was larger than 10^−10^ T/m and by visual inspection.

After artifact rejection, data epochs were band-pass filtered to five distinct frequency bands using 6th-order elliptic filters. The pass-bands were 2–4 Hz, 4–8 Hz, 8–17 Hz, 17–34 Hz, and 35–70 Hz. After filtering, the data were downsampled to approximately six times the highest frequency component included in each filter. Each signal was first forward and then backward filtered to eliminate phase distortion.

### Nested synchrony analysis

**Figure 2:**
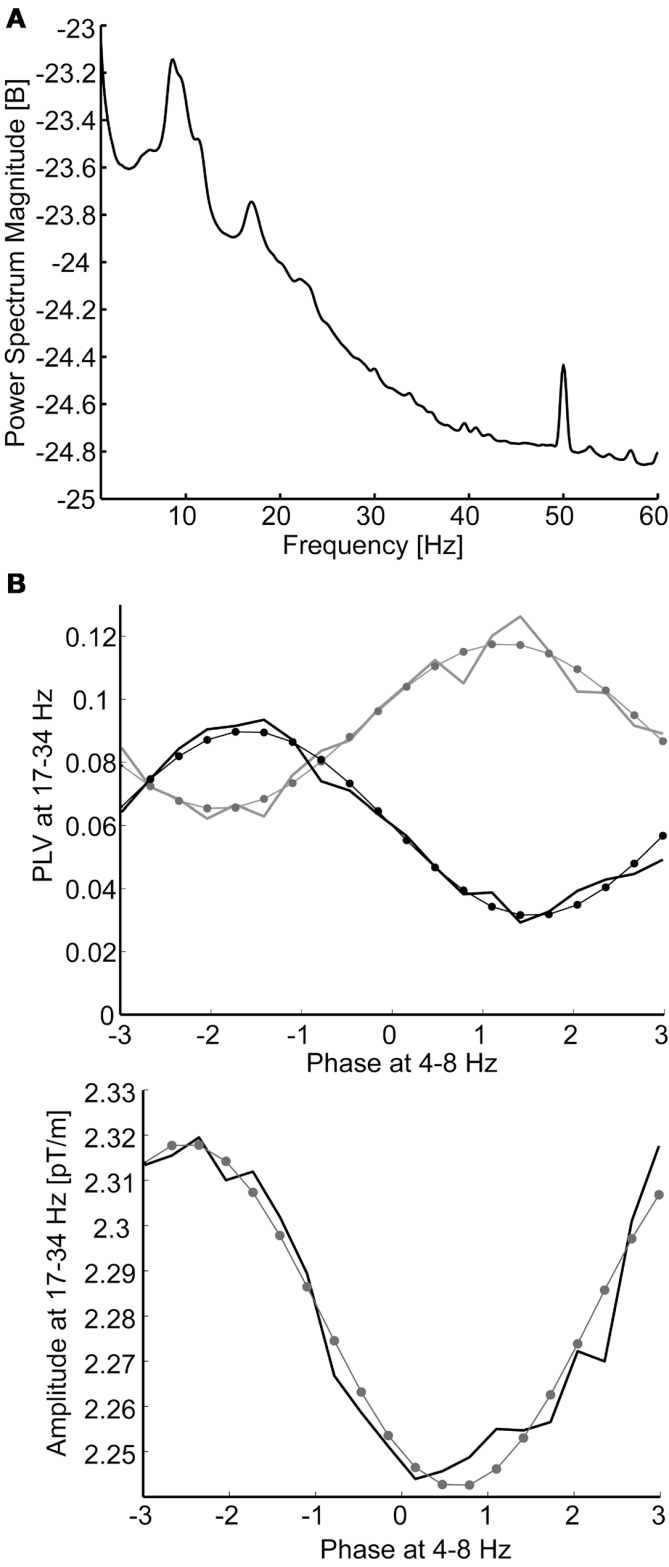
**(A)** The grand average power spectrum pooled over all gradiometer channels and subjects. Only the 8–17 Hz frequency band coincides with peaks in the spectrum. **(B)** Modulation of beta-band (17–34 Hz) phase synchrony (above) and oscillation amplitude (below) as a function of theta-band (4–8 Hz) phase. Data are sorted to 20 phase bins and modeled with sinusoidal fits to the data (thin lines with dots at bin centers). Data are from a single subject: nested synchrony of MEG channel 190 with channels 16 (black line) and 35 (gray line), illustrating antagonist synchrony modulation, and nested oscillation in channel 190. The adjusted R^2^ goodness-of-fits were 0.97 and 0.95 (above) and 0.95 (below).

To find out if the MEG data showed nested synchrony between two frequencies, *f*_X_ < *f*_Y_, the phase locking value (Lachaux et al., 1999) between data from two gradiometer channels, *x*_i_ and *x*_j_, at frequency *f*_Y_ was computed in 20 bins. The bins were determined by the phase of *x*_i_ at frequency *f*_X_, ϕ^i^_X_, so that each bin included 5% of the samples—thus, the amount of data was uniform across the bins. The continuous phase of the signal *x*_i_ in frequency *f*_X_, or *x*^i^_X_, was computed with its Hilbert transform (**H**) as ϕ^i^_X_ = arg[**H**(*x*^i^_X_)], where arg(*x*) is the argument, or phase, of a complex-valued *x*. Because estimation of PLV (phase-locking value) in short time windows suffers from high variance, we first computed the phase difference time series between channels *x*_i_ and *x*_j_ at frequency *f*_Y_: Δϕ^i,j^_Y_ = arg[**H**(*x*^i^_Y_)**H**(*x*^j^_Y_)^*^], where ^*^ denotes complex conjugate. Then, each phase difference sample was sorted to one of the 20 bins according to the concurrent phase of *x*_i_ at frequency *f*_X_, ϕ^i^_X_. After sorting the phase difference data to phase bins, PLV was computed within each bin as PLV^i,j^_Y_ = abs[Σ exp(*i*Δϕ^i,j^_Y_)]/*N*, where *i* = (−1)^1/2^ and *N* is the number of samples in one bin. The result from this procedure is the higher-frequency PLV in the 20 consecutive bins of the lower oscillation phase range (Figure 2B). Then, a non-uniform PLV distribution would signify nested synchrony between channels *x*_i_ and *x*_j_ and between frequencies *f*_X_ and *f*_Y_. We characterized the non-uniformity, or modulation, of the PLV distribution by fitting a sinusoidal period *a*_i,j_ × sin[{ϕ^i^_X_} + *f*_i,j_] + *b*_i,j_ to the PLV data; here, *a*_i,j_ is the magnitude of sinusoidal modulation of synchrony between *x*_i_ and *x*_j_, *f*_i,j_ is the phase shift of the sine function, *b*_i,j_ is the constant term (roughly equal to the mean PLV across all bins), and {ϕ^i^_X_} are the centers of the 20 phase bins of ϕ^i^_X_. The sinusoidal fit was adopted to ensure that possible non-uniformity of the distribution was not due to stochastic fluctuations. In addition, the modulation was expected to be 2π-periodic, at most one peak or trough was expected in the distribution, and the model is simple (two non-trivial parameters, *a*_i,j_ and *f*_i,j_). The sine model was found to be acceptable by visual inspection of the data and the degree-of-freedom-adjusted R^2^ goodness-of-fit values (see Figure 2). This nested synchrony analysis procedure was repeated for all pairs of channels (204 channels) and for frequency pairs that were not adjacent (six pairs). The sampling frequency in nested analysis was roughly six times of the highest frequency component in the higher frequency band data.

Nested amplitude modulation was analyzed in the same way as nested synchrony, except that the amplitude *A* of the single channel *x*_i_ in the higher frequency band *f*_Y_, instead of the phase difference of two channels, was estimated using Hilbert transform, *A*^i^_Y_ = abs[**H**(*x*^i^_Y_)], and averaged in the bins determined by the phase of the lower-frequency oscillation, ϕ^i^_X_.

To ensure that the possible findings of nested synchrony are not due to complicated data processing, similar pre-processing and data analyses were performed for noise data, which were recorded in a magnetically shielded room where no subject was present.

### Statistical evaluation

The significance of individual sinusoidal fits was checked by estimating the 95% confidence interval for the sinusoidal modulation amplitude, *a*_i,j_, and inspecting that the confidence interval did not include 0. Significance of nested synchrony was then evaluated by generating 100 sets of surrogate data. These were created by permuting the order of epochs when choosing the phase bins from the lower-frequency data, while keeping the phase difference data itself intact. The real PLV data were then *z*-transformed (by subtracting the mean and dividing by standard deviation of the surrogate PLV values) to see if it differed significantly from the surrogate data. We used Bonferroni-corrected α = 0.05 as the level of significance. The number of tests was *n* = 204 × 203 = 41,412, so corrected level of significance was α_*n*_ = α/*n* = 1.21 × 10^−6^. The *z*-score required for a significant nested synchrony was then obtained from the cumulative standard normal distribution at the value 1-α_*n*_, resulting in *z* = 4.7. In the case of nested oscillation, or amplitude modulation, the number of tests is *n* = 204, and the level of significance became *z* = 3.5.

### Simulations of cross-frequency coupling

We simulated two time series to represent recordings of neuronal activity at two distinct channels. The aim of these simulations was to inspect if nested oscillations and nested synchrony can be regulated independently, under varying levels of noise. The recorded signals were simulated with 10,000 samples of white noise, which was then filtered to two distinct frequency bands with a 5-fold frequency difference. The effects from nested interactions were then simulated by making the amplitude (in the case of nested oscillations) or the phase (in the case of nested synchrony) of the faster oscillations correlate with the phase of the slower oscillation. Separate parameters controlled the strength of nested oscillations, nested synchrony, and noise level. We then analyzed the resulting signals for nested oscillations and nested amplitude, like explained above (section “Nested Synchrony Analysis”).

## Results

### Presence of nested oscillations in resting-state MEG data

**Figure 3:**
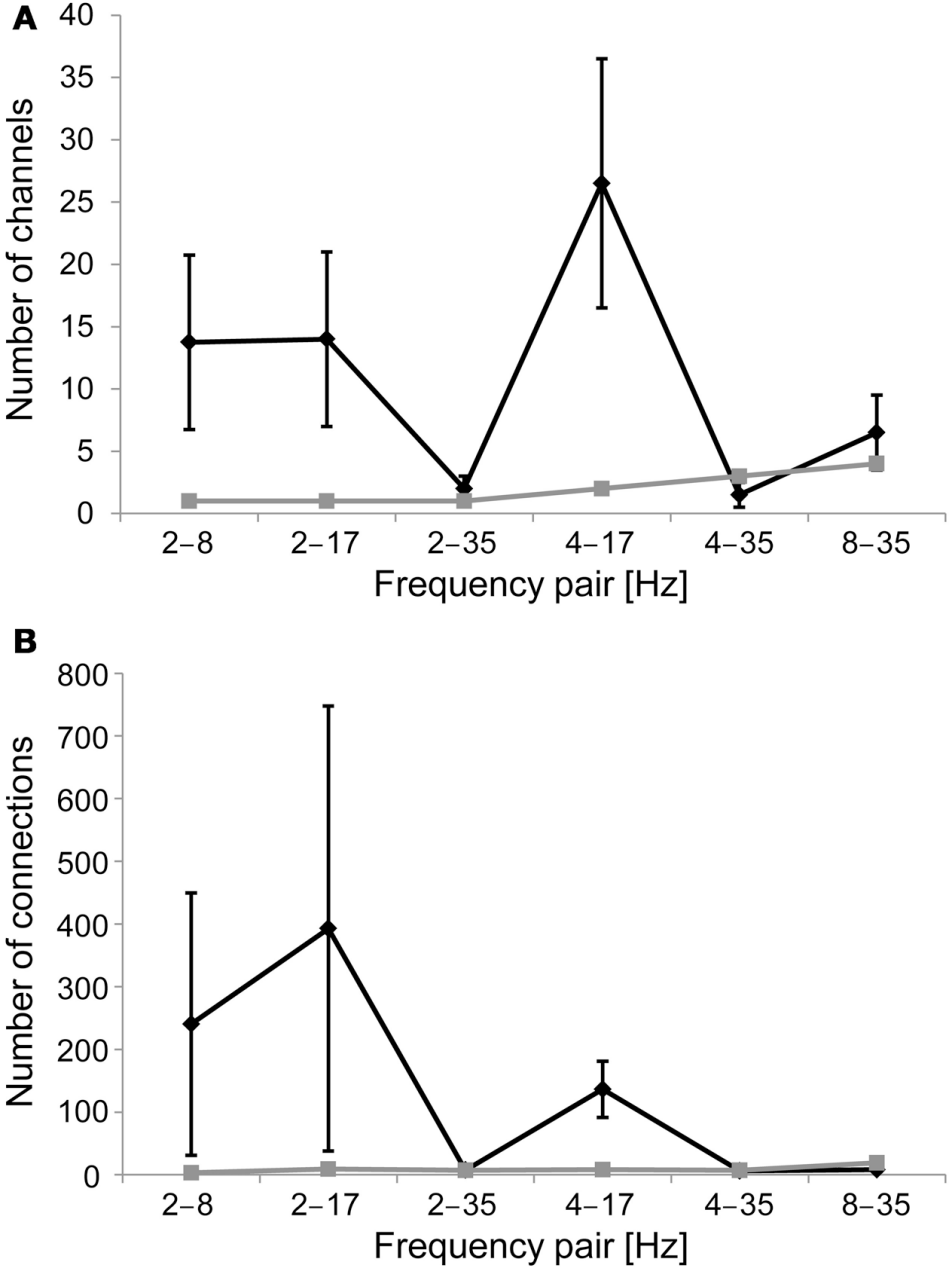
**(A)** Number of channels that display significant nested oscillations (mean ± S.E.) in each frequency band pair. The black line is for the mean over subjects, and the gray line is for empty-room data. **(B)** As in **(A)**, but the number of channel pairs that display significant nested synchrony in each frequency band pair is indicated.

We first aimed at replicating earlier findings of nested oscillations. We evaluated the presence of cross-frequency amplitude modulations, or nested oscillations, in each gradiometer channel by computing the mean amplitude of a higher frequency oscillation in 20 bins determined by the phase of a lower frequency oscillation, by fitting a sinusoid to those data, and then comparing the amplitude modulation to that found in 100 sets of shuffled surrogate data and empty-room data (see Methods). The mean number of channels with significant nested oscillations (*z* > 3.5; Bonferroni correction with *n* = 204 and *p* < 0.05) per subject and frequency pair was 11 for the real data, whereas it was only 2 for the empty-room data (Figure 3A). Nested oscillations were the most prominent between frequency pairs 2–8 Hz, 2–17 Hz, and 4–17 Hz.

### Presence of nested synchrony in resting-state MEG data

We evaluated cross-frequency modulation of a higher-frequency phase synchrony as a function of the phase of a slower oscillation, or nested synchrony, between all MEG gradiometer channel pairs. We computed PLV in 20 bins, which were determined by the phase of the slower oscillation, estimated the sinusoidal modulation over these bins, and confirmed the statistical significance of observed effects using a surrogate distribution from 100 sets of shuffled data, as well as empty-room data (see Methods). The mean number of channel pairs with significant nested synchrony (*z* > 4.7; Bonferroni correction with *n* = 204 × 203 and *p* < 0.05) per subject and frequency pair was 132 for the real data, whereas it was only 9 for the empty-room data (Figure 3B). Nested synchrony was the strongest between frequency pairs 2–8 Hz, 2–17 Hz, and 4–17 Hz. Although statistically significant nested synchrony was found in the data, it was present only in a small fraction of channel pairs. There was, on average, less than one significant connection per gradiometer channel after correction for multiple comparisons. We then checked if the real data were better fitted with the sinusoidal function than the surrogate data by inspecting the number of significant sinusoidal fits. As expected, this number was generally higher for the real data than for the shuffled data (grand average *z*-score = 2.6), and individually significant (*z* > 2.32, corresponding to *p* < 0.01) in 9 out of 24 subject-frequency pairs. The sinusoidal fits of original empty-room data were not significantly better than those of shuffled empty-room data (mean *z*-score = 0.9). These findings provide evidence for nested synchrony in human brain activity.

### Nested synchrony and changes in oscillation amplitude

**Figure 4:**
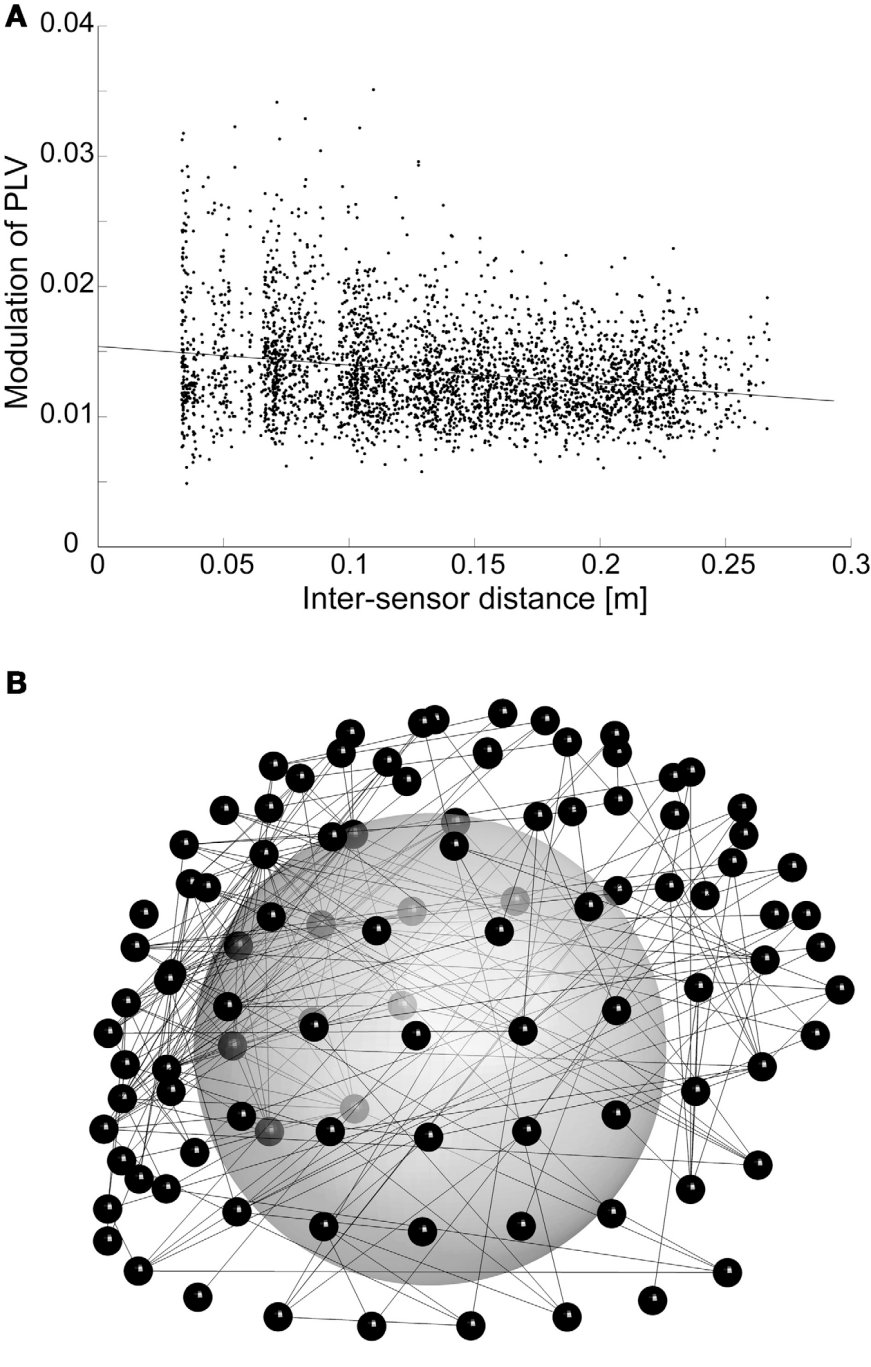
**(A)** The modulation of PLV among higher-frequency (17 Hz) oscillations by the phase of an oscillation at lower frequency (4 Hz) is plotted against inter-sensor distance. This subject and frequency pair was selected, because it had the strongest correlation between distance and nested synchrony strength. For clarity, we visualize only those nested synchrony data that are significant and have adjusted R^2^ goodness-of-fit higher than 0.75 (3418 data points). **(B)** Spatial visualization of the data in **(A)**, but with R^2^-criterion of 0.9 (308 connections). The connections (lines) are depicted between sensors (small spheres) that are visualized in their 3D positions. The large sphere demonstrates the head inside the MEG helmet; the occipital pole is in the lower left corner, nose pointing to the right.

Phase synchrony among recording channels is known to be sensitive for artefacts due to volume conduction. Although we use planar gradiometer sensors with local sensitivity profiles to reduce this effect, there is still some artefactual contribution. However, the fact that we are inspecting modulation of synchrony reduces the vulnerability of our results to volume conduction. With fixed sources, the analyses are affected only when the amplitude of oscillations changes, which leads to changing patterns and magnitudes of artefactual synchrony. Furthermore, PLV estimates may be affected by two potential mechanisms of amplitude-caused bias: either higher oscillation amplitudes lead to enhanced SNR (signal-to-noise ratio) for oscillations, which then causes higher PLV estimates for the same underlying neuronal synchrony, or signals with low amplitude can become buried under common-form noise, which may then lead to higher noise-induced synchrony between those channels. Taken that nested oscillations, or amplitude modulation by low-frequency phase, has been established previously and was reproduced here, nested synchrony could potentially be related to such amplitude effects. However, there are several findings that point to a different direction. First, the relative modulation of amplitude is smaller than the relative modulation of phase synchrony (*p* < 10^−10^, *t*-test across all significant connections in each subject and frequency pair), and it is not conceivable that small amplitude changes would cause relatively larger changes in phase synchrony. Furthermore, the preferred phases of amplitude and synchrony modulation are not the same: although linear regression between the preferred phases of amplitude and synchrony suggest significant correlation (*p* < 0.001 for all significant connections in each subject and frequency pair), the dependency is very weak (mean slope = 0.06). This means that amplitude and synchrony are enhanced at distinct times of the oscillatory cycle, thus the changes in oscillation amplitude via nested oscillations could not cause the nested synchrony observed here. Finally, it is established that artefactual synchrony due to volume conduction and measurement geometry is concentrated to the shortest inter-sensor distances. We found that the modulation of synchrony by lower-frequency phase often decreases as a function of inter-sensor distance, but the effect is very small (Figure 4A): it explains at most 1% of variability in the data (mean slope = −0.003). Furthermore, connectivity patterns typical for synchrony generated by volume conduction are not apparent in the spatial reconstructions (Figure 4B). Together, these analyses suggest that nested synchrony observed in this study is not an artifact due to volume conduction.

### Nested oscillations and nested synchrony are independent

To confirm that nested oscillations in two channels can be regulated independently of their nested synchrony, we performed simulations of two time series that were coupled via these cross-frequency relationships with different signal-to-noise levels (see section “Simulations of Cross-Frequency Coupling”). These simulations showed that it is possible to vary each of the three interactions simultaneously without affecting the others (Table 1).

**Table 1:** **Simulations of two coupled time series show that nested oscillations and nested synchrony can be controlled independently**.

**amp1**	**amp2**	**sync1**	**nestAmp1**	**nestAmp2**	**nestSync1**	**SNR**
0	0	0	0,002	0,003	0,013	∞
1	0	0	0,623	0,005	0,017	∞
0	0	1	0,002	0,005	0,279	∞
1	1	1	0,628	0,612	0,287	∞
0	0	0	0,003	0,006	0,011	5
1	0	0	0,610	0,006	0,021	5
0	0	1	0,004	0,007	0,277	5
1	1	1	0,610	0,588	0,275	5
0	0	0	0,010	0,013	0,013	1
1	0	0	0,440	0,015	0,024	1
0	0	1	0,010	0,012	0,156	1
1	1	1	0,439	0,420	0,220	1

Changing the parameters controlling coupling within each signal (nested oscillations, amp1 and amp2) or coupling between the signals (nested synchrony, sync1) only affects the interaction (nestAmp1, nestAmp2, and nestSync1) that parameter is controlling. Changing the level of noise (SNR) does not affect the results remarkably.

## Discussion

We have introduced and tested a novel cross-frequency interaction model of nested synchrony. In this model the neuronal inter-areal oscillatory interactions are modulated by a lower-frequency oscillation, in an analogous fashion to nested oscillations discovered previously (Lakatos et al., 2005; Canolty et al., 2006; Monto et al., 2008; Osipova et al., 2008; He et al., 2010). Our data indicate that nested synchrony is robustly, although sparsely, present in extracranial recordings of human brain activity. Nested synchrony was present in several frequency pairs, but it turned out that there was practically no nested synchrony or nested amplitude modulation in the gamma-band. This is probably because of poor SNR of gamma-band oscillations (Figure 3A). Another explanation could be that inter-areal gamma-band synchrony is difficult to observe consistently with scalp recordings, as high-frequency synchronization is often attributed to short-range neuronal communication.

There are some potential caveats in our analyses of nested synchrony. PLV as a measure of oscillatory phase synchrony is sensitive not only to genuine phase correlations but also to artefacts from volume conduction and/or field spread. There are, however, grounds to believe that volume conduction does not play a significant role here. Our analysis has internal control for such artifacts, because we are not characterizing patterns of synchrony directly but the modulation of synchrony by the phase of a slower oscillation. Thus, artefactual synchrony could only play a role if the faster oscillatory amplitudes were modulated by the slower oscillation in the same fashion. However, we found that the relative modulation of synchrony was larger than the relative modulation of amplitude, and that the preferred phases of synchrony and amplitude were not identical (see section “Nested Synchrony and Changes in Oscillation Amplitude”). In addition, the lengths of nested synchrony connections (Figure 4A) and their spatial patterns (Figure 4B) support the view that volume conduction is not causing the nested synchrony found in this study, although its effect cannot be entirely neglected. Another possible caveat is the rather complicated data analysis methodology, which could produce some unintended effects. To rule out the possibility that nested synchrony would arise as an artifact of data processing, we recorded empty-room data, where no neuronal activity is present, and subjected these data to the same analyses than the subject data. We found no evidence of nested synchrony in the empty-room data (Figure 3B). This absence of nested synchrony in the absence of a subject suggests that recorded neuronal activity underlies the observed nested synchrony. Finally, it can be suspected if nested modulation of amplitude and phase can take place simultaneously in two signals. We addressed this issue by performing simulations of two time series, and modulated these cross-frequency interactions parametrically. Our simulations indicate that it is possible to independently control the modulation of amplitude within two signals and the modulation of phase synchrony between them, although underlying physiological mechanisms are not reached with these simulations (Table 1).

Nested oscillations have been investigated in many studies previously. Perhaps the most popular subject in this field has been short-term memory, where the idea of temporal segmentation of memory contents by nested oscillations has been proposed (Lisman and Idiart, 1995). In a more recent line of research, the functional significance of nested oscillations in perception and attention has been elucidated, and the nested relationships are proposed to mediate a coupled hierarchy of oscillation frequencies (Lakatos et al., 2005, 2008). Furthermore, robust and widespread nested temporal relationships were discovered in arrhythmic (non-oscillatory) data as well (He et al., 2010), which might indicate the presence of fractal organization in brain background activity. Yet, complementary to studies above, modulation of spatial patterns of nested oscillations according to experimental tasks has been described, suggesting that these patterns may play a role in cognitive operations (Canolty et al., 2006).

Our results support the idea that phase synchrony dynamics are not regulated in isolation for each brain region and frequency band, but are intimately linked to neuronal oscillations in other brain regions and frequency bands. The findings also suggest that oscillatory inter-areal synchronization may be coordinated in varying time scales. If there are, indeed, oscillations in several frequency bands that contribute to changes in oscillation synchrony via the mechanism of nested synchrony, they may together play a significant role in dynamically coordinating the strength of interactions between oscillatory neuronal ensembles. This idea would consolidate the view of brain function being composed of hierarchically coupled scales (Lakatos et al., 2005; Palva and Palva, 2011). However, nested relations have been found even between non-oscillatory, or arrhythmic, activities (He et al., 2010). If the same holds true for nested synchrony as well, there arises a possibility for scale-free modulation of neuronal synchrony through the entire continuum of temporal and spatial scales. It must be noted here that the frequency ratio is not a limiting factor in the formulation of nested synchrony. In the current study, only a limited selection of frequency pairs was inspected. More detailed analysis would be required to determine if nested synchrony exists specifically between a set of narrow frequency bands or if it generalizes over several frequencies, including those where no peak in the amplitude spectrum can be seen.

While studies of nested oscillations have been successful in elucidating cross-frequency relationships in neuronal oscillations, the notion of nested synchrony proposed here could extend and corroborate these findings by combining cross-frequency interactions to inter-areal synchrony. Of particular interest here are the studies on the cross-frequency model of working memory, where slow (theta) oscillations phase controls faster (gamma) oscillations to store memories in their temporal patterns (Lisman and Idiart, 1995; Jensen and Lisman, 2005). As it is known that both theta and gamma oscillations participate in mediating information between hippocampal regions as well as between hippocampus and neocortex (Sirota et al., 2008; Colgin et al., 2009; Colgin, 2011), it would be interesting to see if nested modulations could be the mechanism for keeping the complex dynamics of multi-frequency oscillations and interactions in the hippocampo-neocortical system organized. Intriguingly, tight synchronization between hippocampal and cortical neuronal spikes has been linked to the theta oscillation generated in the hippocampus in rats (Siapas et al., 2005). Along similar lines, cortical gamma-band coherence was found to be correlated with hippocampal theta oscillations (Sirota et al., 2008). These data offer a putative example of rhythmically occurring inter-areal synchrony that is mediated by a slower oscillation, partially validating the idea of nested synchrony in a more detailed scale. The synchronization of intrinsic rhythmical activities in the brain to rhythmic external stimuli and related enhancement in stimulus processing (Schroeder and Lakatos, 2009) also point toward nested synchrony, because attention and processing of stimulus features are often promoted by synchronization of high-frequency oscillations. Another interesting and related example can be found in processing of speech: there, a coordinated hierarchy of feature processing levels and timescales is needed to execute and integrate the multitude of sub-tasks that are required to comprehend all aspects of speech (Hickok and Poeppel, 2007). Indeed, there exist interesting data on theta-entrained phase coding and spatio-temporally distributed processing of speech stimuli (Luo and Poeppel, 2007; Giraud et al., 2007). It remains to be seen if processing of speech is organized by nested relationships within and between specialized processing streams.

Local excitability changes are thought to underlie nested oscillation amplitude modulations, due to mechanisms related to either neuronal network properties or local environmental conditions (Jensen and Colgin, 2007; Lakatos et al., 2008). However, nested synchrony does not follow straightforwardly from local excitability changes, which have been related to slow oscillations, because it specifically requires coordination of phases among the higher-frequency activities. Even tight correlation between the slow oscillations is not an adequate condition for nested synchrony in the higher frequency band, unless there is direct *n*:*m* phase locking. On the mechanistic side, nested oscillations and nested synchrony need not be entirely separate phenomena, because they both are related to changes in neuronal synchrony: whereas enhanced inter-areal synchrony can often be deciphered with scalp recordings, enhanced local synchrony is effectively seen as increased oscillation amplitude. The neuronal basis of phase-accurate synchronization over a distance is currently under investigation, and it might rely on different cellular mechanisms than synchronization over short distances (Kopell et al., 2000). The generation mechanisms of nested synchrony depend on the neuronal mechanisms that establish and sustain oscillation synchrony in the first place. Interneuron networks have been credited a central role in neuronal synchronization, and their properties might change depending on the phase of the slower oscillation. However, interneuron projections are mostly local, so this modulation would be expected to affect the local oscillations instead of long-distance synchrony. A more plausible mechanism could thus be related to long-range pyramidal cell projections, where changes would allow local oscillations to continue as driven by the interneuron network but would affect long-distance synchronization (Kopell et al., 2000). Here, pyramidal cell membrane conductances would be the favored target for modulations by the slow oscillation.

We have so far investigated solely how slow oscillations in one area modulate phase synchrony between that area and another one. Yet, even more complex patterns may emerge from nested interactions. First, the individual pair-wise inter-areal synchronies are probably a part of at least one larger network. Second, sub-networks may in turn be regulated by different phases of distinct slower oscillations. Third, synchronization among the slower oscillations could provide another means to integrate different networks, all of which may carry different functionalities for information processing. These possibilities demonstrate the potential versatility of nested effects in mediating relationships between oscillatory activities in the brain, as well as the high number of possible combinations of cross- and within-frequency oscillatory interactions in brain dynamics.

In this article, we have described the model of nested synchrony, validated its existence in human neuronal activity, and proposed that it could be a viable candidate for mediating interactions between oscillatory networks at different frequencies and separated neuronal populations. In particular, it offers the possibility for a local neuronal network to participate in distinct neuronal interactions through simultaneously active mechanisms using phase-based coding only. In future, intracranial recordings will be needed to shed light on the extent and more detailed features of nested synchrony. Furthermore, this model could be applied to further investigate the interrelations between very slow brain activities, as observed with fMRI or full-band EEG, and faster neuronal oscillations (Monto et al., 2008). In addition, the subject's state could be manipulated experimentally to assess the functional significance of nested synchrony.

### Conflict of interest statement

The author declares that the research was conducted in the absence of any commercial or financial relationships that could be construed as a potential conflict of interest.
